# Activity of ampicillin-sulbactam, sulbactam-durlobactam, and comparators against *Acinetobacter baumannii-calcoaceticus* complex strains isolated from respiratory and bloodstream sources: results from ACNBio study

**DOI:** 10.1128/aac.00379-25

**Published:** 2025-07-17

**Authors:** Ecem Buyukyanbolu, Jill Argotsinger, Eric T. Beck, Robin R. Chamberland, Andrew E. Clark, Anne R. Daniels, Rachael Liesman, Mark Fisher, Philip Gialanella, Jonathan Hand, Amanda T. Harrington, Romney M. Humphries, Holly Huse, Robert Hamilton-Seth, Julia D. Hankins, Wesley D. Kufel, Scott W. Riddell, Jamie Marino, Lars F. Westblade, A. Brian Mochon, Navaneeth Narayanan, Thomas J. Kirn, Virginia M. Pierce, Raghava Potula, Tsigereda Tekle, Patricia J. Simner, Robert J. Tibbetts, Christine Vu, Lilian M. Abbo, Octavio Martinez, Rebekah E. Dumm, David P. Nicolau, Tomefa E. Asempa, on behalf of the Acinetobacter baumannii Complex National Biorepository (ACNBio) Study Group

**Affiliations:** 1 Center for Anti-Infective Research and Development, Hartford Hospital 23893, Hartford, Connecticut, USA; 2 Advocate Lutheran General Hospital 21886, Park Ridge, Illinois, USA; 3 Department of Microbiology, ACL Laboratories 300396 , Milwaukee, Wisconsin, USA; 4 Department of Pathology, Saint Louis University School of Medicine 12274, St. Louis, Missouri, USA; 5 Department of Pathology, University of Texas Southwestern Medical Center 465773, Dallas, Texas, USA; 6 Froedtert & Medical College of Wisconsin 37353, Milwaukee, Wisconsin, USA; 7 Department of Pathology, Froedtert & Medical College of Wisconsin 37353, Milwaukee, Wisconsin, USA; 8 Wisconsin Diagnostic Labs 655052, Milwaukee, Wisconsin, USA; 9 Department of Pathology, University of Utah School of Medicine 12348, Salt Lake City, Utah, USA; 10 Associated Regional and University Pathologists (ARUP) Laboratories 33294, Salt Lake City, Utah, USA; 11 Department of Pathology, Albert Einstein College of Medicine, Montefiore Medical Center 2013, New York, New York, USA; 12 Department of Infectious Diseases, Ochsner Health 633467 , New Orleans, Louisiana, USA; 13 Loyola University Medical Center 25815, Maywood, Illinois, USA; 14 Department of Pathology, Microbiology and Immunology, Vanderbilt University Medical Center 204907, Nashville, Tennessee, USA; 15 Department of Pathology, Harbor-UCLA Medical Center 21640, Torrance, California, USA; 16 Department of Pathology and Laboratory Medicine, University of Kansas Medical Center 21638, Kansas City, Kansas, USA; 17 State University of New York Upstate University Hospital, Syracuse, New York, USA; 18 Binghamton University School of Pharmacy and Pharmaceutical Sciences 465824, Binghamton, New York, USA; 19 State University of New York Upstate Medical University, Syracuse, New York, USA; 20 Department of Pathology and Laboratory Medicine, Weill Cornell Medicine 12295, New York, New York, USA; 21 Division of Infectious Diseases, Department of Medicine, Weill Cornell Medicine 12295, New York, New York, USA; 22 Banner Health 3605, Phoenix, Arizona, USA; 23 Sonora Quest Laboratories 273991 , Phoenix, Arizona, USA; 24 Department of Pathology, University of Arizona College of Medicine 42283 , Phoenix, Arizona, USA; 25 Department of Pharmacy Practice and Administration, Ernest Mario School of Pharmacy, Rutgers University 15484 , Piscataway, New Jersey, USA; 26 Department of Pathology & Laboratory Medicine, Rutgers Robert Wood Johnson Medical School 12287 , New Brunswick, New Jersey, USA; 27 Department of Pathology, University of Michigan Medical School 12266 , Ann Arbor, Michigan, USA; 28 Pathology and Laboratory Medicine, Temple University Health System 12315, Philadelphia, Pennsylvania, USA; 29 Department of Pathology, Johns Hopkins University School of Medicine 1500, Baltimore, Maryland, USA; 30 Pathology and Laboratory Medicine, Henry Ford Health 2971, Detroit, Michigan, USA; 31 Department of Pharmacy, Jackson Health System 23214, Miami, Florida, USA; 32 Department of Medicine, University of Miami Miller School of Medicine 12235, Miami, Florida, USA; 33 Department of Pathology and Immunology, Washington University School of Medicine 12275, St. Louis, Missouri, USA; 34 Division of Infectious Diseases, Hartford Hospital 23893, Hartford, Connecticut, USA; Shionogi Inc., Florham Park, New Jersey, USA

**Keywords:** *Acinetobacter*, sulbactam, CRAB, sulbactam-durlobactam

## Abstract

Infections caused by carbapenem-resistant *Acinetobacter baumannii-calcoaceticus* complex (ABC) are associated with high mortality rates and limited treatment options. This study aims to evaluate the *in vitro* activity of clinically utilized antimicrobials against a contemporary collection of ABC isolates with a predominant carbapenem-resistant phenotype. Geographically dispersed US medical centers (*n* = 22) provided non-duplicate respiratory and bloodstream ABC isolates for surveillance testing. Antimicrobial susceptibility testing was conducted by broth microdilution and interpreted according to Clinical & Laboratory Standards Institute (CLSI) and Food and Drug Administration (FDA) breakpoints. ABC isolates (*n* = 523) from respiratory tract (74.4%) and blood (25.6%) sources were recovered from patients (2023–2024). Forty percent were obtained from intensive care unit patients. Carbapenem non-susceptibility was observed in 76.9% of isolates and was more common among respiratory tract cultures. The addition of durlobactam to sulbactam decreased the MIC_90_ by three-doubling dilutions from 32 to 4 µg/mL, increasing the susceptibility rate to 96.9% from 33.8%. Genome sequencing of sulbactam-durlobactam non-susceptible isolates (16/523; *n* = 3.1%) revealed MBL and non-enzymatic resistance mechanisms. Cefiderocol inhibited 93.5% and 76.1% of isolates at CLSI and FDA susceptible breakpoints, respectively. Minocycline susceptibility was <50%, while tigecycline and eravacycline MIC_50/90_ were 1/2 and 0.5/1 µg/mL, respectively. Sulbactam-durlobactam displayed high activity against sulbactam (95.4%), carbapenem (96.3%), and cefiderocol (95.2%) non-susceptible isolates. Susceptibility rates of clinically utilized antimicrobials against a US collection of ABC isolates ranged from 23% to 97%, with meropenem displaying the lowest rate and sulbactam-durlobactam demonstrating the highest overall rate. Sulbactam-durlobactam activity was preserved against sulbactam, carbapenem, and cefiderocol non-susceptible isolates among respiratory tract and bloodstream isolates.

## INTRODUCTION

*Acinetobacter baumannii-calcoaceticus* complex (ABC) isolates are responsible for serious infections such as bacteremia and healthcare-associated pneumonia (1, 2). These infections, typically nosocomial in nature, are associated with high mortality rates and extended hospital lengths of stay (3, 4). Until recently, the available treatment options for ABC included high-dose ampicillin-sulbactam, carbapenems, polymyxins, high-dose tetracyclines, and cefiderocol. However, these agents are associated with potentially higher toxicity profiles, poor pharmacokinetics, increasing resistance, or limited clinical data (5–7). These challenges led to the development and FDA approval of a β-lactam-β-lactamase inhibitor combination agent (sulbactam-durlobactam) to treat hospital-acquired bacterial pneumonia and ventilator-associated bacterial pneumonia caused by susceptible strains of ABC (7–10).

Sulbactam is an Ambler Class A β-lactamase inhibitor as well as a β-lactam with intrinsic antibacterial activity against *Acinetobacter* species, mainly due to inhibition of penicillin-binding proteins 1 and 3 (PBP1 and PBP3, respectively), which are essential components of cell wall synthesis (11). Durlobactam is a β-lactamase inhibitor with a modified diazabicyclooctane (DBO) scaffold with a broader spectrum of activity compared to other DBO inhibitors such as avibactam. In particular, the inhibition by durlobactam of Class D β-lactamases (OXA-23, OXA-24/40), which are largely responsible for ampicillin-sulbactam and carbapenem resistance, makes durlobactam a β-lactamase inhibitor well suited for targeting ABC infections (7, 9–12).

With the rising incidence of carbapenem-resistant *A. baumannii* (CRAB) and lack of head-to-head comparison of commonly utilized antibiotics including cefiderocol in the United States, an *Acinetobacter baumannii-calcoaceticus* Complex National Biorepository (ACNBio) was established in 2023. This multicenter national surveillance program allows the phenotypic and genotypic characterization of clinical ABC strains collected from diverse geographical regions within the United States.

## MATERIALS AND METHODS

### Participating medical centers

The microbiology laboratories of 22 geographically diverse medical centers across the United States were selected to participate in this study. All sites received local institutional review board approval or waiver prior to participation. Sites provided de-identified data: date of collection, patient sex, age, location at time of culture (intensive care unit [ICU] vs non-ICU), and specimen source.

### Bacterial isolates

ABC isolates were collected from blood and respiratory tract sources between 2023 and 2024. For this study, respiratory tract sources included expectorated or induced sputum, tracheal aspirates, Lukens trap secretions, bronchoalveolar lavage fluid, and protected specimen bushes of the lower respiratory tract. Isolates were identified by the microbiology laboratories of each participating medical center via conventional automated identification methods including the VITEK (bioMérieux) and BD Phoenix Automated Microbiology System (Becton Dickinson), MicroScan (Beckman Coulter), as well as by matrix-assisted laser desorption ionization time-of-flight mass spectrometry (VITEK MS, bioMérieux; Bruker Biotyper, Bruker Daltonics). Duplicate isolates, defined as any isolate of the same species collected from the same patient during the patient hospital encounter, regardless of the susceptibility profile, body source, or specimen type, were excluded. After local culture identification, isolates were transferred to Trypticase soy agar (TSA) slants (Becton Dickinson, Sparks, Maryland) and shipped to the Center for Anti-Infective Research and Development (CAIRD) (Hartford, CT) for susceptibility testing. At CAIRD, isolates were subcultured onto TSA with 5% sheep blood plates (Becton Dickinson, Sparks, Maryland), and frozen in skim milk at −80°C until susceptibility testing was conducted.

### Antimicrobial susceptibility

Durlobactam (Corden Pharma #138677) and eravacycline (Tetraphase #0026000135) powders were supplied by Innoviva Specialty Therapeutics. Ampicillin (MedChemExpress #81549), sulbactam (USP #R03980), meropenem (Sigma Aldrich #LRAD1014), cefiderocol (Fetroja commercial vial #0021), colistin (MedChemExpress #62961), tigecycline (Sigma Aldrich #LRAC6890), and minocycline (Sigma Aldrich #191077) antibiotic powders were purchased. MIC broth trays were prepared with a Biomek 3000 (Beckman Instruments, Inc., Fullerton, CA) across an MIC range specific to each antibiotic: ampicillin/sulbactam (0.125/0.06 to 128/64 µg/mL), sulbactam/durlobactam (0.125/4 to 128/4 µg/mL), meropenem (0.06 to 64 µg/mL), cefiderocol (iron-depleted broth; 0.031 to 32 µg/mL), colistin (0.016 to 16 µg/mL), tigecycline (0.016 to 16 µg/mL), eravacycline (0.016 to 16 µg/mL), and minocycline (0.016 to 16 µg/mL). Susceptibility tests were conducted by manual broth microdilution and interpreted according to CLSI M100 standards (13, 14). Sulbactam MICs were interpreted using the sulbactam component of ampicillin/sulbactam (CLSI breakpoint 8/4 µg/mL) and thus assessed with a sulbactam susceptibility breakpoint of 4 µg/mL. Cefiderocol MICs were assessed with both CLSI (4 µg/mL) and FDA (1 µg/mL) susceptibility breakpoints. *A. baumannii* NCTC 13304, *Escherichia coli* ATCC 25922, and *Pseudomonas aeruginosa* ATCC 27853 were used as quality control strains for antimicrobial testing and run in parallel with test isolates on each day.

### Whole genome sequencing and analysis

Molecular contributors to reduced susceptibility in isolates with sulbactam-durlobactam ≥8 mg/L were investigated. Extraction of chromosomal DNA, whole genome sequencing (WGS), and analysis were performed by Element Materials Technology (North Liberty, IA, USA). Briefly, total genomic DNA was extracted and purified in a KingFisher Flex Magnetic Particle Processor (Thermo Scientific). Total genomic DNA was sequenced on a NextSeq 1000 Sequencer (Illumina, San Diego, California, USA) and NextSeq 1000/2000 P2 Reagents (300 cycles). The DNA libraries for the NextSeq 1000 Sequencer were prepared using the Illumina DNA library construction protocol and index kit (Illumina).

An in-house designed software was used to target assembled sequences as queries to align against PBPs sequences derived from *A. baumannii* ATCC 17978, the sequences of efflux pumps AdeNIJK, AdeRSAB, and AdeLFGH derived from *A. baumannii* ATCC 17978, and a curated database of b-lactamase resistance determinants drawn from the NCBI Bacterial Antimicrobial Resistance Reference Gene Database. Potential matches were generated for each sample following the criteria of >94% identity and 40% minimum coverage length. Initial matches were analyzed further. Sequences displaying 100.0% homology with the reference sequences were named according to the reference. Genes with homology <100.0% were curated and annotated as the gene name showing the closest homology, and amino acid alterations were recorded.

Isolates were epidemiologically typed through multi-locus sequence typing (MLST) using the Institute Pasteur scheme. MLSTs were confirmed by extracting previously defined sets of seven housekeeping gene fragments (∼500 bp). Each fragment was compared to known allelic variants for each locus (housekeeping gene) using the MLST database (PubMLST) available at https://pubmlst.org.

### Analysis

Demographic data were assessed using descriptive statistics including percentages for categorical data. Susceptibility rates (percentage of susceptible isolates within the total number of isolates), cumulative susceptibility plots (number of isolates at each MIC), MIC_50_, and MIC_90_ were computed using Microsoft Excel and presented for each antibiotic, where applicable.

## RESULTS

During the 2023–2024 surveillance period, 523 unique ABC isolates from respiratory and bloodstream sources were collected and evaluated in this study. The demographic data of the isolates are presented in Table 1. The median patient age was 60 years, and 37.1% of patients were female. Most isolates (74.4%) were recovered from a respiratory tract source, and ICU patients contributed 41.5% of all isolates.

**TABLE 1:** Demographics and characteristics of the study population^*c*^

Description	Population (*N* = 523)
Age, median (IQR), years	60 (48–70)
Female sex, *n* (%)	194 (37.1%)
Site location^*a*^	
Northeast	134 (25.6%)
South	121 (23.2%)
Midwest	259 (49.5%)
West	9 (1.7%)
Patient location	
ICU	217 (41.5%)
Medical ICU	132
Surgical ICU	37
Other ICU^*b*^	48
Non-ICU	298 (57%)
NA	8 (1.5%)
Culture source	
Respiratory	389 (74.4%)
Bloodstream	134 (25.6%)

^*a*^ U.S. Census Bureau Regional Divisions.

^*b*^ Other ICU: Burn, neurology, cardiac.

^*c*^ ICU, intensive care unit; NA, not available.

The results of antimicrobial susceptibility testing and MIC_50/90_ values are shown in Table 2. Carbapenem non-susceptibility (NS) was observed in 402 isolates (76.9%). Most carbapenem-NS isolates were from a respiratory source (*n* = 327; 81.3%), compared with bloodstream isolates (*n* = 75, 18.7%). Among all isolates tested, sulbactam-durlobactam was the most active agent (96.9%) followed by cefiderocol (93.5% per CLSI; 76.1% per FDA). Sulbactam displayed an MIC_50/90_ of 16/32 µg/mL and a susceptibility rate of 33.8%. The activity of sulbactam-durlobactam was similar for isolates collected in the ICU and non-ICU settings. Sulbactam-durlobactam maintained high activity against sulbactam-NS (95.4%), carbapenem-NS (96.3%), and cefiderocol-NS (95.2%) isolates (Table 3). As displayed in the cumulative frequency distribution of sulbactam and sulbactam-durlobactam in Table 4, the addition of durlobactam lowered the MIC_90_ three-doubling dilutions, from 32 to 4 µg/mL. Of the 16 isolates (3.1%) that demonstrated sulbactam-durlobactam MIC values ≥ 8 µg/mL, 12 underwent WGS (Table 5). Sequence type two was the predominant MLST identified after molecular typing. All sequenced strains encoded genes for *Acinetobacter*-derived cephalosporinase (ADC) Ambler class C β-lactamase such as ADC-73, as well as various Ambler class D OXA β-lactamases (OXA-23, OXA-58, OXA-66 carbapenemases). A New Delhi metallo-β-lactamase (NDM) was identified in four isolates. Target site alterations in PBP3 and mutations in the AdeIJK efflux system and its transcriptional regulator adeN were noted in the majority of isolates (Table 5).

**TABLE 2:** Antimicrobial susceptibility rates and MIC_50/90_ values of ABC isolates isolated from respiratory, bloodstream, ICU, and non-ICU sources

Category	Antimicrobial agent	%S	%I	%R	MIC_50_ (µg/mL)	MIC_90_ (µg/mL)
All isolates (*n* = 523)	Sulbactam^*a*^	33.8	13.8	52.4	16	32
	Sulbactam-durlobactam	96.9	1.9	1.2	2	4
	Meropenem	23.1	0.2	76.7	64	128
	Cefiderocol_CLSI_	93.5	1.1	5.4	0.5	4
	Cefiderocol_FDA_	76.1	7.6	16.3	0.5	4
	Colistin	–^*c*^	94.1	5.9	0.5	1
	Tigecycline^*b*^	–	–	–	1	2
	Eravacycline^*b*^	–	–	–	0.5	1
	Minocycline	47.8	7.6	44.6	2	8
Respiratory isolates (*n* = 389)	Sulbactam^*a*^	26.2	14.9	58.9	16	32
	Sulbactam-durlobactam	96.9	2.1	1.0	2	4
	Meropenem	15.9	0.3	83.8	64	128
	Cefiderocol_CLSI_	92.5	1.3	6.2	0.5	4
	Cefiderocol_FDA_	73.0	9.3	17.7	0.5	4
	Colistin	–	94.1	5.9	0.5	1
	Tigecycline^*b*^	–	–	–	2	2
	Eravacycline^*b*^	–	–	–	0.5	1
	Minocycline	42.1	6.2	51.7	4	16
Bloodstream isolates (*n* = 134)	Sulbactam^*a*^	56.0	10.4	33.6	4	32
	Sulbactam-durlobactam	97.0	1.5	1.5	1	4
	Meropenem	44.0	0.0	56.0	32	128
	Cefiderocol_CLSI_	96.3	0.7	3.0	0.25	4
	Cefiderocol_FDA_	85.1	3.0	11.9	0.25	4
	Colistin	–	94.0	6.0	0.5	1
	Tigecycline^*b*^	–	–	–	1	2
	Eravacycline^*b*^	–	–	–	0.25	1
	Minocycline	64.2	11.9	23.9	0.5	8
ICU isolates (*n* = 217)	Sulbactam^*a*^	29.0	12.9	58.1	16	32
	Sulbactam-durlobactam	96.8	1.8	1.4	2	4
	Meropenem	18.4	0.5	81.1	64	128
	Cefiderocol_CLSI_	91.7	1.4	6.9	0.5	4
	Cefiderocol_FDA_	71.9	7.8	20.3	0.5	4
	Colistin	–	94.5	5.5	0.5	1
	Tigecycline^*b*^	–	–	–	2	2
	Eravacycline^*b*^	–	–	–	0.5	1
	Minocycline	41.0	8.3	50.7	4	8
Non-ICU isolates (*n* = 298)	Sulbactam^*a*^	37.3	14.4	48.3	8	32
	Sulbactam-durlobactam	97.0	2.0	1.0	2	4
	Meropenem	26.9	0.0	73.1	32	128
	Cefiderocol_CLSI_	95.0	0.7	4.3	0.5	4
	Cefiderocol_FDA_	78.9	7.7	13.4	0.5	4
	Colistin	–	94.0	6.0	0.5	1
	Tigecycline^*b*^	–	–	–	1	2
	Eravacycline^*b*^	–	–	–	0.5	1
	Minocycline	53.7	7.4	38.9	1	8

^*a*^ Sulbactam MICs are interpreted using the sulbactam component of ampicillin-sulbactam MIC breakpoints (≤8/4 [susceptible], 16/8 [intermediate], ≥32/16 [resistant]) per CLSI M100 (Ed35; 2025).

^*b*^ MIC interpretative criteria are not published by CLSI M100 (Ed35; 2025) for tigecycline and eravacycline against *Acinetobacter* spp.

^*c*^ –, not available.

**TABLE 3:** Antimicrobial susceptibility rates and MIC_50/90_ values of relevant phenotypic subsets

Category	Antimicrobial agent	%S	%I	%R	MIC_50_ (µg/mL)	MIC_90_ (µg/mL)
Sulbactam non-susceptible isolates (*n* = 346)	Sulbactam^*a*^	0.0	20.8	79.2	16	32
	Sulbactam-durlobactam	95.4	2.9	1.7	2	4
	Meropenem	4.3	0.3	95.4	64	128
	Cefiderocol_CLSI_	90.2	1.7	8.1	0.5	8
	Cefiderocol_FDA_	69.9	8.4	21.7	0.5	8
	Colistin	–^*c*^	92.8	7.2	0.5	1
	Tigecycline^*b*^	–	–	–	2	2
	Eravacycline^*b*^	–	–	–	0.5	1
	Minocycline	33.0	7.8	59.2	4	16
Carbapenem non-susceptible isolates (*n* = 402)	Sulbactam^*a*^	17.6	16.4	66.0	16	32
	Sulbactam-durlobactam	96.3	2.2	1.5	2	4
	Meropenem	0.0	0.2	99.8	64	128
	Cefiderocol_CLSI_	91.5	1.5	7.0	0.5	4
	Cefiderocol_FDA_	69.4	10.0	20.6	0.5	4
	Colistin	–	93.8	6.2	0.5	1
	Tigecycline^*b*^	–	–	–	2	2
	Eravacycline^*b*^	–	–	–	0.5	1
	Minocycline	33.8	9.5	56.7	4	16
Cefiderocol_FDA_ non-susceptible isolates (*n* = 125)	Sulbactam^*a*^	16.8	13.6	69.6	16	64
	Sulbactam-durlobactam	95.2	1.6	3.2	2	4
	Meropenem	1.6	0.0	98.4	64	128
	Cefiderocol_FDA_	0.0	32.0	68.0	4	64
	Colistin	–	88.0	12.0	0.5	4
	Tigecycline^*b*^	–	–	–	2	4
	Eravacycline^*b*^	–	–	–	0.5	1
	Minocycline	53.6	16.8	29.6	1	8

^*a*^ Sulbactam MICs are interpreted using the sulbactam component of ampicillin-sulbactam MIC breakpoints (≤8/4 [susceptible], 16/8 [intermediate], ≥32/16 [resistant]) per CLSI M100 (Ed35; 2025).

^*b*^ MIC interpretative criteria are not published by CLSI M100 (Ed35; 2025) for tigecycline and eravacycline against *Acinetobacter* spp.

^*c*^ –, not available.

**TABLE 4:** Cumulative frequency distributions of sulbactam and sulbactam-durlobactam MICs against all isolates^*a*^

Antimicrobial agent	Cumulative % of isolates inhibited at MIC (µg/mL) of:										
	≤0.125	0.25	0.5	1	2	4	8	16	32	64	>64
Sulbactam	0.2	0.2	2.7	12.6	23.7	33.8	47.6	75.7	**93.7**	98.9	100
Sulbactam-durlobactam	3.1	7.6	23.1	45.7	78.0	**96.9**	98.9	99.2	99.2	99.6	100

^*a*^ Bolded values represent ≥90% isolate inhibition.

**TABLE 5:** Characteristics of the 12 sulbactam-durlobactam non-susceptible isolates that were subject to whole genome sequencing (WGS)

Isolate	Culture source	Sulbactam MIC (μg/mL)	Sulbactam-durlobactam MIC (μg/mL)	MLST^*a*^	PBP1a variant	PBP1b variant	PBP2 variant	PBP3 variant	Beta-lactamase genes	Efflux genes AdeNIJK^*b*^
1	Respiratory	64	16	2	–	P112S	–	A515V	ADC-73, OXA-23, OXA-66	AdeN[K57I], AdeJ[K847E]
2	Respiratory	32	8	2	–	P112S	–	–^*c*^	ADC-56, OXA-66, OXA-72	AdeN[Y100X], AdeJ[K847E]
3	Bloodstream	32	8	2	–	P112S	–	–	ADC-56, OXA-66, OXA-72	AdeJ[K847E]
4	Bloodstream	32	16	2	–	P112S	–	A515V	ADC-73, OXA-23, OXA-66	AdeJ[K847E]
5	Respiratory	16	8	499	–	–	P665A	T511S	ADC-222, OXA-23[M1X], OXA-24, OXA-95	AdeN[C87fs_K98X], AdeI[A400T], AdeJ[K847E], AdeK[P264S]
6	Bloodstream	>64	64	570	–	P112S	–	A515V	ADC-73, OXA-23, OXA-66[T10K], NDM-1, TEM-1	AdeJ[K847E]
7	Respiratory	8	8	499	–	–	P665A	–	ADC-222, OXA-23, OXA-24, OXA-95	AdeN[C87fs_K98X], AdeI[A400T], AdeJ[K847E], AdeK[P264S]
8	Respiratory	>64	128	2	–	P112S	–	A515V	ADC-73, OXA-23, OXA-66, NDM-5, TEM-1	AdeJ[K847E]
9	Respiratory	16	8	2	–	P112S	–	A515V	ADC-73, OXA-23, OXA-66	AdeN[S45fs_Q91X], AdeJ[K847E]
10	Respiratory	>64	128	2	–	P112S	–	A515V	ADC-73, OXA-23, OXA-66, NDM-5, TEM-1	AdeJ[K847E]
11	Respiratory	32	8	2	–	P112S	–	A515V	ADC-73, OXA-23, OXA-66	AdeN[S45fs_Q91X], AdeJ[K847E]
12	Respiratory	>64	64	10	A219T	A626T	I108, G340R, P665A	–	ADC-76, OXA-58, OXA-68, NDM-1	AdeN[L35fs_D42X], AdeJ[K847E]

^*a*^ Pasteur scheme.

^*b*^ AdeIJK (efflux pump); AdeN (repressor).

^*c*^ –, not identified.

Of the three tested tetracyclines, only minocycline has evaluable CLSI breakpoints resulting in 47.8% susceptibility. Tigecycline and eravacycline inhibited 90% of isolates at MICs of 2 and 1 µg/mL, respectively. With no CLSI susceptible breakpoint, 93.9% of isolates were identified as intermediate to colistin.

## DISCUSSION

The *A. baumannii* national biorepository was established to allow systematic *in vitro* assessment of antimicrobials including the recently approved sulbactam-durlobactam against contemporary isolates from the United States. All the drugs tested in this study have been listed as potential therapeutic agents to treat CRAB infections in the Infectious Diseases Society of America (IDSA) antimicrobial resistance expert guidance (6). Of these agents, sulbactam-durlobactam demonstrated the highest rates of susceptibility against the sulbactam- and meropenem-resistant isolates in this study, supporting its role as a preferred agent in the guidance document (6). Furthermore, the overlap between sulbactam- and carbapenem-resistance, as well as an elevated sulbactam MIC_90_ suggests little utility in a high-dose ampicillin-sulbactam regimen as a treatment option (15, 16).

Two notable studies have also assessed the *in vitro* activity of sulbactam-durlobactam against *Acinetobacter* spp. First, McLeod et al. evaluated 1,722 global clinical ABC isolates (2016–2017) and observed that the MIC_50/90_ values were 1/2 µg/mL, with 97.7% of isolates demonstrating susceptibility to sulbactam-durlobactam (17). In the second study conducted by Karlowsky on 5,032 ABC clinical isolates collected from 33 countries (2016–2021), the MIC_50/90_ values for sulbactam-durlobactam were determined to be 1/2 µg/mL, inhibiting 98.3% of the isolates at the 4 µg/mL breakpoint (18). In both studies, 50% of isolates were carbapenem-resistant compared with 77% in this current study.

Sulbactam-durlobactam retained >95% activity across patient location (ICU vs non-ICU) and infection source (respiratory vs bloodstream). On the other hand, comparator agents displayed higher resistance rates to ABC recovered from ICU patients versus non-ICU patients. The difference in resistance rates to comparator agents was more marked when stratified by infection source (respiratory tract infection isolates being more resistant than bloodstream isolates) and has been reported in other surveillance studies (19).

Cefiderocol does not have harmonized CLSI and FDA MIC breakpoints against *A. baumannii,* thus cefiderocol susceptibility can vary widely based on the institutional practice as evident from the range of susceptibility rates (76.1–93.5%) observed in this current study. Also, the recent CLSI revision of minocycline susceptibility breakpoints from 4 to 1 µg/mL results in a <50% susceptibility rate and dampens the clinical use of minocycline for ABC. Across all isolates, tigecycline and eravacycline MICs tended to be onefold and twofold lower than minocycline, respectively. Further evaluation of tigecycline and eravacycline is complicated by lack of FDA and CLSI breakpoints against *A. baumannii*. However, in a similar trend to minocycline, both tigecycline and eravacycline MIC_50/90_ against ABC were comparable to that shown against *Enterobacterales* (20, 21).

In this study, the overall resistance rate of sulbactam-durlobactam was 1.2%. This rate, generated with a US data set is similar to the 2% resistance rates reported in the literature for global isolate collections (7). Sulbactam-durlobactam resistance mechanisms included metallo-β-lactamase production and mutations in PBP3 and efflux pumps. Similar findings have been observed in surveillance studies with NDM and PBP3 alterations as the predominant contributors to decreased sulbactam-durlobactam susceptibility (22–24).

A noteworthy observation from this study is that while multidrug-resistant pathogens are often regarded as problematic in the ICU population, the majority of carbapenem-NS ABC isolates were recovered from non-ICU patients. This trend has been reported for *P. aeruginosa* and warrants resource allocation for stewardship and infection control screening outside the ICU (25). An exploration of risk factors is also needed with previous factors for carbapenem resistance among *P. aeruginosa* such as carbapenem use, presence of medical devices, previous hospitalization/long-term care visit, likely contributing to carbapenem-resistant ABC as well (26, 27).

In conclusion, the contemporary respiratory and bloodstream ABC strains within this biorepository demonstrate a wide range of susceptibilities (23%–97%) to clinically utilized antimicrobials, with meropenem displaying the lowest rate and sulbactam-durlobactam demonstrating the highest rate. Sulbactam-durlobactam activity was preserved against sulbactam-, carbapenem-, and cefiderocol-NS isolates and across infection sources, highlighting its role in the treatment of multi-drug-resistant *A. baumannii* infections. Continued local, regional, and global surveillance studies are needed to track susceptibility rates and inform epidemiology, stewardship, and infection control efforts.
